# Support provided by midwives to women during labour in a public hospital, Limpopo Province, South Africa: a participant observation study

**DOI:** 10.1186/s12884-018-1860-8

**Published:** 2018-06-05

**Authors:** Maria S. Maputle

**Affiliations:** Department of Advanced Nursing, University of Venda, Private Bag X5050, Thohoyandou, 0950 South Africa

**Keywords:** Support offered, Labour pains, Communication between women and midwives, Informational support, Emotional support, Physical comforting measures

## Abstract

**Background:**

Physical presence during labour offer women opportunity of having positive childbirth experiences as well as childbirth outcomes. The study aimed to determine what support provided by midwives during intrapartum care at a public hospital in Limpopo Province. The study was conducted at a tertiary hospital in Limpopo Province.

**Methods:**

A participant observation approach was used to achieve the objectives of the study. The population comprised of all women who were admitted with labour and for delivery and midwives who were providing midwifery care in the obstetric unit of a tertiary public hospital in Limpopo Province. Non-probability, purposive and convenience sampling were used to sample 24 women and 12 midwives. Data were collected through participant observations which included unstructured conversations with the use of observational guide, field notes of all events and conversations that occurred when women interact with midwives were recorded verbatim and a Visual Analog Scale to complement the observations. Data were analysed qualitatively but were presented in the tables and bar graphs.

**Results:**

Five themes emerged as support provided by midwives during labour, namely; communication between women and midwives, informational support, emotional support activities, interpretation of the experienced labour pain and supportive care activities during labour.

**Conclusion:**

The communication between woman and midwife was occurring as part of midwifery care and very limited for empowering. The information sharing focused on the assistive actions rather than on the activities that would promote mothers’ participation. The emotional support activities indicated lack of respect and disregard cultural preferences and this contributed to inability to exercise choices in decision-making. The study recommended the implementation of Batho Pele principles in order to provide woman-centred care during labour.

**Electronic supplementary material:**

The online version of this article (10.1186/s12884-018-1860-8) contains supplementary material, which is available to authorized users.

## Background

The Batho-Pele Principles in the White Paper introduced a customer-focused approach with the aim of transforming systems, procedures, attitudes and behaviour within the childbirth units and re-orient midwives in the customers’ favour, an approach which put people first [1]. In this study, the observations were focused on consultation, standard, courtesy and information. These would be evident when women were treated with courtesy and consideration; it was observed whether mothers were allowed to practice their preferences during childbirth (courtesy). The practice in public hospitals, mothers were provided with full and accurate information regarding childbirth process and midwifery and intrapartum care which they were entitled to receive (information). Hence, the researcher conducted the study to determine the support provided by midwives to women during labour at a public hospital in Limpopo Province. Support provision during labour was viewed to be having impact on the childbirth experience as well as on childbirth outcomes [2]. Midwives are there to provide support to women to offer them strength of facing challenge of giving birth without losing control [3, 4].

Provision of support during labour is important and is continuous, so as reduce the risk of medical interventions, like emergency caesarean section or regional analgesia maybe less prevalent and labour maybe shorter [5]. The labour supporter could play a major role in increasing women’s chances to have a positive initial breastfeeding experience. Rosen [6] describes support as an interactive process which may be affected by persons’ age, experience, and personality as well as by the environment [6]. Whereas Simkin [7] views labour support as continuous presence, emotional support that includes encouragement, guidance, and reassurance; physical comforting which was assistance in carrying out coping techniques, use of touch, massage, positioning, and movement) [7]. Simkin further indicate that labour support includes provision of information and guidance to the woman and her partner; and to further assist the woman to express her needs and wishes; and provision of advice, anticipatory guidance, and explanations of procedures [7]. Labour support was viewed as an affordable intervention that responds to the basic emotional and physical needs of a woman during a painful and vulnerable moment of her reproductive carrier – child birth.

The pain during labour is a physiological phenomenon [8]. Ralph et al. [8] further pointed that, sources of pain during the first stage was associated with reduced blood supply to the uterine muscles during contractions. In the second stage, the source of pain was associated with the stretching of vaginal wall and perineum and compression of pelvic structures during the passage of descending head. The pain that women experienced during labour was affected by multiple physiological and psychological factors and its intensity varied greatly.

In studies “*Experiences of mothers of care provided by midwives during childbirth* by Maputle and Nolte [9] and *Experiences of midwives of providing care to women during childbirth”* by Maputle and Hiss [10] it was noted that the labour ward of a tertiary hospital was experiencing shortage of staff and open plan labour ward with no privacy and this had negative impact on the allowing the male partner to support to women during labour. There was shortage of midwives wherein, one midwife would care for more than one woman at the same time. The remarks cited by mother transcripts’ were: *“I couldn’t communicate freely with the midwife because she was very busy, but she examined me when I reported/ requested help. The midwife was busy with other mothers; she was not in my cubicle throughout the childbirth”.* This was supported by the study of Schafheutle et al. [11]., who indicated that inadequate nursing staff in a shift may contribute to lack of time in providing adequate care and this is cited as barrier to effective pain management, by interfering with applying non-pharmacological pain relief methods.

In the same study by Maputle and Nolte [9], authors found that one participant preferred the male partner to be available during childbirth; however, her wish was not honoured because of the physical structure of the ward. Tournaire and Theau-Yonneau [12] pointed out that the availability of doula to provide support was considered an important part of natural pain relief methods. Leeman et al., [13] advocated for the effective use of conventional approaches to emphasize the interaction between mind, body and environment [13]. Lack of social and emotional support from partner/ doula or excessive medical intervention to women during labour is factors that may be related to increased intensity of pain [14]. Labour support could be perceived as the presence of an empathic person who offers information, comforting measures and other forms of tangible assistance to enable a woman cope with the stress of labour and birth. At this public hospital, women were always alone during labour. In South Africa, the provision of midwifery care during labour should be aligned to Batho-Pele Principles, namely; consultation, service standard, courtesy, access, information, openness and transparency, redress and value for money [1]. It was against this background that the study was conducted.

## Methods

### Study design

A qualitative participatory research approach was used to determine the support provided by midwives to women during labour at a public hospital in Limpopo Province.

### Study setting

The study was conducted in the labour ward of a public hospital in Limpopo province, South Africa. The hospital is a 509 bed hospital, out of these beds only 45 were allocated to labouring women. The total number of normal deliveries would be ±10 per day. The total number of midwives who were allocated in the units during the day was twenty (but on duty there would be eight midwives, because some would be on leave or off duty).

### Population and sample

Population comprised of all women, admitted in labour to deliver their babies and the midwives who were providing midwifery care and who consented to participate in the study. Non-probability, convenience and purposive sampling in De Vos et al., [15] were used. Only women who were found to be in labour during the day of data collection were included in the study. The selection of midwife was guided by whether the midwife who is on duty had been already observed when providing care. The inclusion criteria for women were: full term pregnancy (38–42 weeks) and should be in early active phase of labour (cervical dilatation of 3–10 cm, regular uterine contractions), with the presence of the foetal heart beat. All the midwives who had at least two years’ experience in the labour unit who agreed to participate in the study. The researcher sampled women and midwives [16]. The sample comprised of 24 women and 12 midwives.

### Data collection

Data were collected by researcher who is a holder of PhD in Midwifery and Neonatal nursing, during the day shift in August – December 2015. The researcher was also shared the same culture and language with participants. The researcher collected data through participant observations which included semi-structured observational guide, observing the activities, interactions and conversations between the women and the midwives during intrapartum care (Additional file 1). The use of participant observation gave additional and more accurate information on behaviour of people [17]. This semi-structured observation method enabled the researcher to describe events and behaviour as they occurred during labour [17]. Observations were limited to the interaction between a woman and a midwife during labour and the midwifery care rendered that is; communication, informational support, emotional support activities, physical comforting measures rendered and supportive care activities when the woman is experiencing labour pains. Observations were carried when a woman was in the active phase of labour, when the cervical dilatation was 3 cm to the delivery of the placenta. The unstructured conversations were held informally with women and midwives throughout labour, because these conversations were spontaneous and emerged out of natural social interaction and contributed to the depth and richness of information that otherwise would have been difficult to capture through more structured interviews. Field notes of events were recorded in a notebook and conversations that occurred when women interact with midwives were recorded verbatim by the use of a voice recorder. To complement the informal conversations, Visual Analog Scale (VAS) which is an instrument and a simple scale was completed by the women and midwives independently during the following dilatations of the cervix 0–3, 4–7 and > 8 cm. Recording of field notes form a core of record [17]. It entailed both the empirical observations and their interpretation.

### Data analysis

Biographical data of both women and midwives and the data obtained through the unstructured observation guide, the VAS were analysed through frequency distribution and the data were presented as percentages in tables and bar graphs and clustered into 5 themes [18]. The field notes and informal voice conversations were analyzed qualitatively through open coding and were mergered with the 5 themes.

### Ethical considerations

Ethical clearance was obtained from the Ethics Committee of the University of Venda (SHS/11/PDC/001) and permission to access the health facility and conduct the study from the Provincial Department of Health. The principles of informed consent, beneficence and right to privacy were observed. Participation in the study was voluntary and no remuneration was given. The consent of participants was obtained before data collection.

## Results

### Theme 1: Communication between women and midwives during labour

**Fig. 1:**
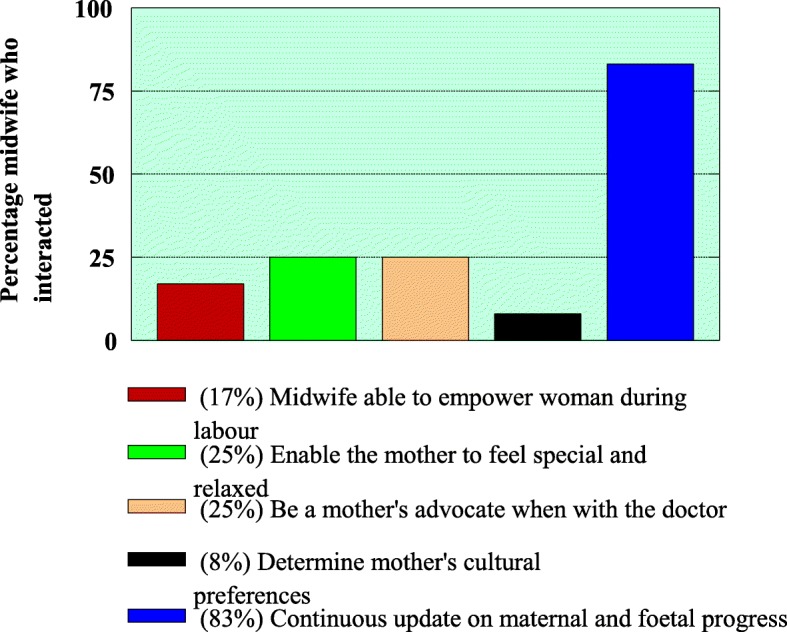
Interaction/ communication between women and midwives

Communication between women and midwives during labour was determined by observing the interactions and activities as presented in Fig. 1:

- Ability of midwife to empower the woman
- Enabling the woman to feel special and relaxed (by explaining the physiological changes and encouraging relaxation exercises)
- Advocacy skill
- Determining the woman’s cultural and personal preferences
- Continuous updating on foetal and maternal progress

The observation showed that (10) 83% of midwives where continuously updated the mother on maternal labour progress and on foetal well-being. Limited affective communication was displayed which was thought to facilitate mutual participation as evidenced by (3) 25% of midwives advocating mothers when with the doctor, (2) 17% of the midwives were able to empower mothers, (3) 25% of the midwives were enabling the mother to feel special and relaxed and (1) 8% of the midwives determined the mother’s cultural/personal preferences. Some of the direct quotes from participants that indicate limited empowering during labour were *“my midwife was not telling me what is happening and I think she must be patient and at least listen to what I want to say”.* With regard to determining the mother’s cultural preferences, one participant said *“Hmm … I didn’t clearly understand the language of the midwife”,* so this indicated that cultural preferences were not determined. Another participant said “*if I indicate the cultural preference she didn’t allow or even listen to why I was making such a preference.*”

### Theme 2: Informational support during childbirth

**Fig. 2:**
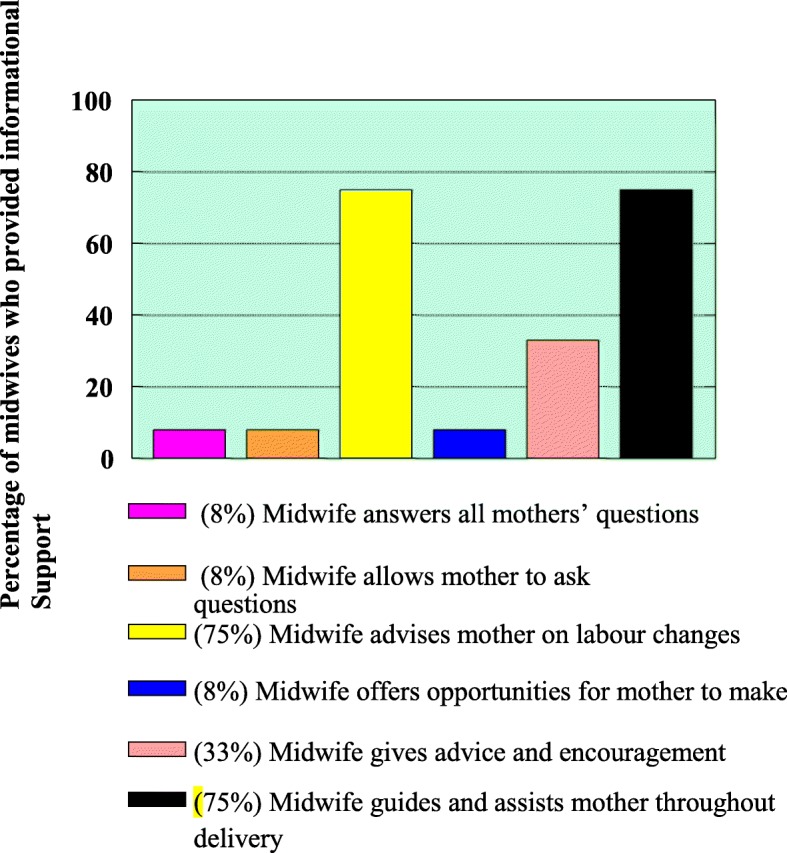
Informational support and exchange between mothers and attending midwives

From the profile of mothers recruited for the study (Table 1), 23 (96%) did not participate in childbirth preparation classes. Only 1 (4%) mother participated in childbirth classes, hence it can be inferred that mothers probably had limited information support from the attending midwives during childbirth. To determine whether informational support was given by the attending midwives to mothers during childbirth, the observed informational support as presented in Fig. 2, namely;

- Answering of mother’s questions
- Allowing/encouraging mothers to ask questions
- Advising mothers on the physiological changes of labour
- Offering mothers opportunities to come up with suggestions
- Extending advice and encouragement
- Guiding and assisting mothers throughout delivery

**Table 1:** Demographic profile of mothers and midwives recruited for the study

Mothers	*N* = 24	%	Midwives	*N* = 12	%
1. Age in years on your last birthday			1. Gender		
16–20	7	29	Male	0	0
21–30	12	50	Female	12	100
31+	05	21			
2. Parity:			2. Qualifications		
Primigravida	13	54	Registered midwife	10	83
Para 2–3	07	29	Advanced midwife	02	17
Para 4+	04	17			
3. Delivery outcome (exclude fetal deaths			3. Duration of allocation in labour ward - years		
Normal vaginal delivery	19	79	2–4	06	50
Forceps or vacuum extraction delivery	0	0	5–6	04	33
Caesarian section (not elective)	05	21	> 7	02	17
4. Duration of labour (hours)			4. Cultural/ Ethnic group		
2–4	2	8	Northern Sotho	9	75
5–8	4	17	Tsonga	2	17
> 9	18	75	Venda	1	8
			Others	0	0
5. Pain relief during labour			5. Religious affiliation		
Pharmacological	03	13	Protestants	4	33
Non-pharmacological	21	87	Apostolic church	2	17
			Zion Christian church	2	17
			Others	4	33
6. Cultural/ Ethnic group					
Northern Sotho	15	63			
Tsonga	4	17			
Venda	3	12			
Others	2	08			
7. Family status					
Married	10	42			
Single	14	58			
Divorced	0	0			
Widow	0	0			
8. Religious affiliation					
Protestants	14	58			
Apostolic church	3	13			
Zion Christian church	7	29			
Others	0	0			
9. Educational status					
Never literate	2	8			
Primary school literate	5	21			
Secondary school literate	9	38			
Tertiary institution	8	33			
10. Companion present?					
Yes	0	0			
No	24	100			
11. Participation in childbirth preparation classes					
Yes	1	4			
No	23	96			
12. Transfer from another hospital					
Yes	5	21			
No	19	79

It was noted that (9) 75% of the midwives advised mothers on changes taking place during childbirth and guided and assisted them throughout childbirth. Actions that were promoting mothers’ participation were limited as only (4) 33% of the midwives extended encouragements, (1) 8% offered opportunities for mother to give suggestions whilst (1) 8% of attending midwives allowed mothers to ask questions which were answered during childbirth.

The observation was supported by the direct quotes from a labouring woman who said *“my midwife was telling me to breathe in and out but I didn’t know why.”* This showed that midwives are giving advice and encouragement but not advising on the labour changes. However, the other participant said “*Midwives should give me information, and then I would be able to make choices. So as I’m updated with the progress of labour I’m able to make informed choices like relaxation techniques.”*

### Theme 3: Emotional support during childbirth

To determine the emotional support given by the attending midwives to mothers during childbirth, the following were observed:

- Instilling of confidence (with information provided) and encouragement of companion to be present during labour
- Be understanding, friendly and reassuring
- Encouraging free choice and full participation
- Fostering the integration of cultural/personal preferences
- Show of respect

**Fig. 3:**
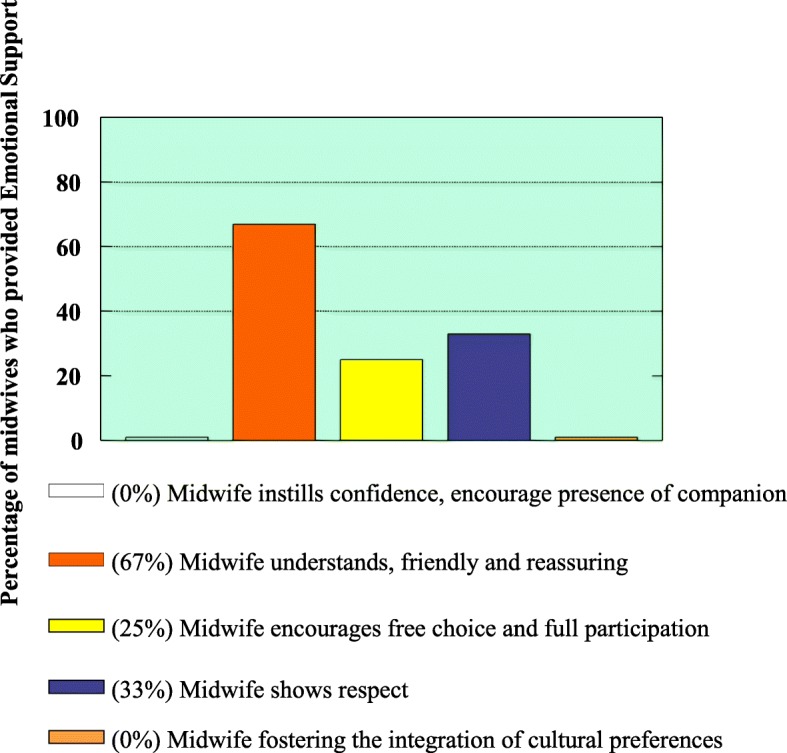
Emotional support activities by attending midwives during labour

It was observed that midwives offered limited emotional support during childbirth as evidenced by the observation that (0) none of the midwives encouraged the presence of a companion and integration of cultural/ personal preferences during childbirth. Only (4) 33% of attending midwives showed respect for mothers during labour whereas (3) 25% were encouraging free choice and full participation of the mothers. A significant number of attending midwives (8) 67% displayed an understanding, friendly and reassuring attitude to mothers during childbirth. Figure 3 illustrates that only (4) 33% of the midwives showed respect for mothers. Some midwives were encouraging participation and this was confirmed by one participant who said “*When I was experiencing severe pain, she told me to lie on the side, sit or adopt any position that I feel comfortable.*” However, contrary to the observed friendliness and reassurance the participant said “*I would also be happy if the midwives can be more approachable, listen to what I say and not to be too harsh.”* The other participant said “*During childbirth I expect midwives to be friendly and to respect me as an adult.”*

### Theme 4: Interpretation of experienced and perceived pain during labour

The visual analog scale (VAS, 100 mm tool) was issued to mothers and their attending midwives with the aim of comparing the pain experienced among mothers during childbirth and to observe the awareness and responses of the attending midwives to the pain exhibited by mothers. Mothers and their attending midwives had to complete the scale independently during the following phases of cervical dilatation:

- 0–3 cm, mothers were experiencing moderate pain while attending midwives perceived as mild pain
- 4–7 cm, mothers experienced severe pain while midwives still perceived this as mild pain
- 8–10 cm, both groups experienced and perceived pain as severe.

**Fig. 4:**
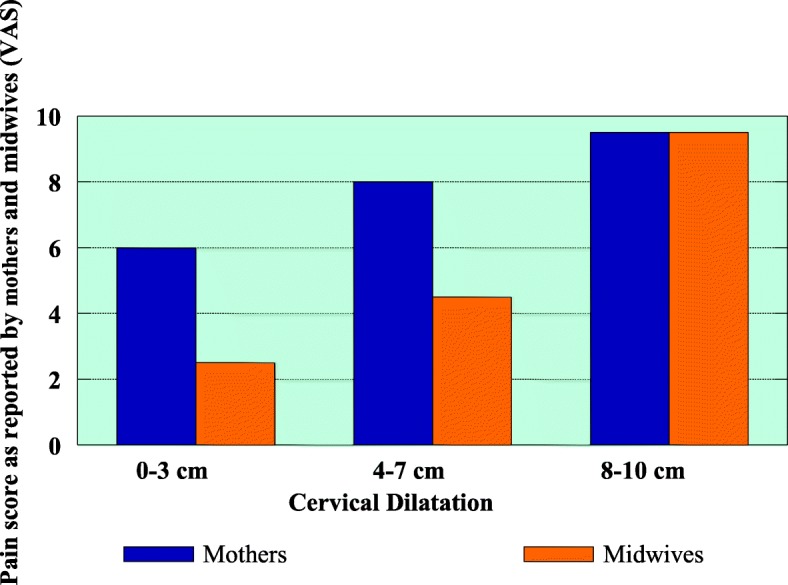
Pain scores by visual analogue scale

All assessments were completed independently and without reference to the previous rating. Findings as presented in Fig. 4.

Findings on VAS on Fig. 4 indicated that mothers were experiencing severe pain throughout labour (with a pain score 6–8), while the pain midwives exhibited (perceived) was mild-moderate at the beginning of labour (2.5–4.5) but at the end of labour, the pain score was similar to that of mothers (9.5).

### Theme 5: Supportive care activities during labour

When the mother was responding to contractions during childbirth, the following supportive care activities were determined:

- Provision of physical care
- Attendance to the elimination needs
- Midwife caring for more than one mother at the same time

**Fig. 5:**
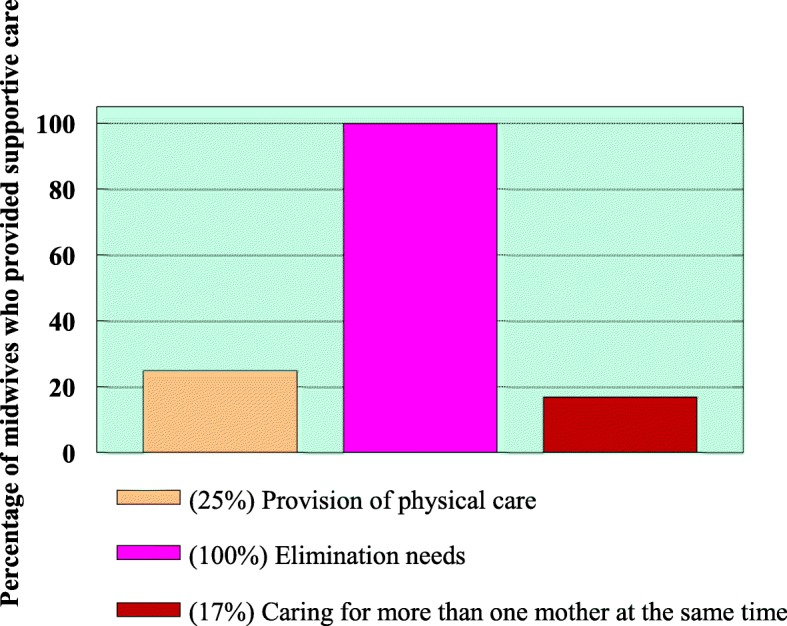
Supportive care activities by attending midwives during labour

Figure 5 presents the findings of supportive care activities by midwives when mothers were experiencing pain.

All midwives provided care for mothers’ elimination needs. Midwives could not give individual care to mothers, as they were caring for more than one mother at the same time. There was a shortage of staff as (2) 17% of the midwives were caring for more than one mother. Only (3) 25% of midwives provided physical care during childbirth (physical care include touching, rubbing/ massaging).

The quotes from participants that supported the observation as cited by one participant was *“the midwife was good to me, because she was assisting me with my elimination needs, hence I was able to deal with my pain.”* However, another woman said “*I am waiting for that other midwife, as the one who was helping me has left”.* For this woman, the midwife was caring for more women.

## Discussion

Five themes emerged as support provided by midwives during labour, namely; communication between women and midwives, informational support, emotional support activities, interpretation of the experienced labour pain and supportive care activities during labour. It was noted that communication between the woman and the midwife occurred more when the midwife was rendering midwifery care and very limited during affective communication. Watkins [19] pointed out that determining women’s preferences for their care in labour is a reasonable basis for caregiving activities. Table 1 indicates that from the total population of mothers (*n* = 24) recruited in the study, 9 (37%) did not share the same language with the attending midwives. Hindered verbal communication as a result of language barriers and non-listening skills were displayed during the interaction of a midwife and a mother during childbirth in this study. Persson and Dykes [20] firmly supposed that the experience of good care appeared to be dependent on communication and behaviour to be able to meet mothers just as they are.

Analysis of the observations in the informational support theme, (Fig. 2) revealed that during information sharing between the mother and the midwife more emphasis was placed on the assistive actions than on the activities that would promote mothers’ participation. Bergstrom et al., [21] indicated that women want information about pain and fear and how to mitigate both, explanations about the instruments and processes involved in birth, and positive communication. The amount and type of information sharing, to include explanations about the process of birth, reports about descent of the foetus’s head, and soliciting information about the woman’s well-being. Bryanton et al., [22] supported that when women have more information about what is happening to and around them and are given options about position changes and timing of pushing, they can take control of the birth.

Rice [23] indicated that lack of respect and disregard of the choices for mothers, in the mothers’ narratives, contribute to apathy towards respect in their treatment and inability to exercise choices in decision-making. Respect empowers women [24]. Results also indicated that none of the midwives encouraged the presence of a companion during childbirth. Brown [25] wrote that the reasons for this restriction may include negative attitude of labour ward staff toward the presence of outsiders in the labour ward. In an incident during the study, one midwife participant was very stressed and insensitive with an instructive attitude. She was also impatient to an extent that little or no explanations were give during the provision of care. Hence, the mother participant was absolutely terrified as no comforting words were forthcoming from the midwife participant. This resulted in mother not co-operating as there was no gestures of understanding between the two.

The VAS is one of the most used pain assessment instruments, both in research and clinical practice [26]. With the VAS, mothers were experiencing severe pain throughout labour while the pain perceived by midwives was mild-moderate. This was supported by Baker et al. [27] who found that midwives were less able to assess pain accurately when women described their pain as severe. The patient’s verbal report is considered to be the single most reliable indicator of how much pain she is experiencing [28]. However, at the end of labour pain score for both mothers and midwives was similar. Pain research indicated that midwives often assess patients’ pain inaccurately [27, 29]. On observation of the midwives’ responses to the mothers’ pain, the responses were similar throughout the childbirth process. The care provided by midwives to the mothers when experiencing pain is displayed in Fig. 5. Very few mothers were given analgesics as prescribed. Hodnett, et al., [30] reinforced that labour support which is non-medical care of a labouring woman, involves physical comforting such as touching, massaging, bathing, grooming, applying warmth or cold Teshome et al., [31] emotional support such as continuous companion, reassurance, encouragement, anticipatory guidance, information provision, and non-medical advice. Whereas Mackey [32]; Lundgren [33] considered praise, flexibility, acceptance, information giving, encouragement, friendliness, presence, confidence, and assisting with breathing and relaxing to be aspects of helpful nursing qualities during labour.

### Limitations

Data was only collected during the day shift from 07 h00 to 18 h00 the support provided by night shift staff could have given different results. The use of participatory observations by semi-structured observational guide may have been influence observer preconceived ideas, which may affect the results of the study.

## Conclusions

The tertiary hospital was challenged with the shortage of staff and this contributed to one midwife to caring for more than one woman at a time. Hence, midwives were mainly providing care for mothers’ elimination needs. The communication was occurring when the midwife was rendering midwifery care and very limited for empowering. The information sharing focused on the assistive actions rather than on the activities that would promote mothers’ participation. The emotional support activities indicated lack of respect and disregard cultural preferences and this contributed to inability to exercise choices in decision-making. Mothers were never given analgesic during labour because the interpretation of experienced and the perceived pain by midwives were different, and mothers were perceived to be in severe pain when the cervical dilatation was 9–10 cm. The presence of companion was not encouraged, hence physical care include touching, rubbing/ massaging were limited. The study recommended the implementation of Batho Pele principles in order to provide woman-centred care during labour.

The recommendations were aimed at improving the standard of the public service and effective service delivery to support women during labour. Services should be based on a customer-orientated framework. These principles were integrated within the framework to provide woman-centered care as follows:

- Midwives are to consult mothers about the level of midwifery care to be received and, where possible, should give (allow) involvement and support choices about the services that are offered (consultation).
- Mothers should be told what level and quality of midwifery care (intervention are to be provided so that they would be aware of what to expect (service standard).
- Mothers are to be treated with courtesy and consideration; they should be allowed to practice their preferences during childbirth (courtesy).
- Midwives should allow all mothers equal access to personal control and decision-making (access).
- Mothers should be given full and accurate information about the childbirth process and midwifery care which they are entitled to receive (information). The Department of Public Service and Administration [1] indicates that the importance of the public service delivery lies in the need to build confidence and trust between the provider (midwife) and the user (mother) through openness and transparency.

Sandall [34] and Midmer [35] were in support of customer-oriented service delivery when they pointed out that the philosophy and focus should shift from technologization to personalization, and to building of the paradigm of woman-centered practice that will be based on equal partnership between mothers and attending midwives.

## Additional file

Additional file 1: Interview guide and Visual Analog Scale (VAS). (DOCX 25 kb)

## Abbreviation

VAS: Visual Analog Scale
