# Supplementary material for: Support provided by midwives to women during labour in a public hospital, Limpopo Province, South Africa: a participant observation study
Source: BMC Pregnancy Childbirth. 2018 Jun 5;18:210. doi: 10.1186/s12884-018-1860-8 (PMC5989402; doi:10.1186/s12884-018-1860-8)
Supplement: Supplementary file 1 — Interview guide and Visual Analog Scale (VAS). (DOCX 25 kb) [file 12884_2018_1860_MOESM1_ESM.docx]

**Additional file 1: INTERVIEW GUIDE**

**SEMI-STRUCTURED PARTICIPANT OBSERVATION GUIDE**

PARTICIPANT (S) : ______________________

DATE AND TIME : ______________________

**Observations were done during the active phase of labour (cervical dilatation of 3 cm with regular contractions) until the end of the third stage of labour is complete.**

**This guide was used to observe the following throughout the childbirth process:**

- **Actors (women and midwives)**
- **Activities/events (interaction between woman and attending midwife during childbirth:**
- **Setting (in the labour ward)**
- **Context (during labour)**

Interaction between women and midwife during labour

| Interaction | Observed | Informal conversations |
| --- | --- | --- |
| **Communication (aspects to observe)** | | |
| Midwife able to empower woman during labour   - Reinforcing the acceptable behaviour during labour - Reinforcing the expected physiological changes that take place |  |  |
| Enable her to feel special and relaxed   - The approach used when meeting the woman - The tone used when communicating to the woman - Adressing the woman by name - Communicating in the woman’s language |  |  |
| Be a woman’s advocate when with the doctor   - Talking for the woman |  |  |
| Determine from woman her cultural/personal preferences |  |  |
| Continuous monitoring of maternal and fetal condition and up-date the woman on progress of labour.   - Updating woman on maternal vital signs in relation to labour, the contractions, cervical dilation, and fetal heart (accelaration and deccelaration |  |  |
| **Informational Support** | | |
| Answer all questions and allow woman to ask questions without feeling that she bothers the midwife |  |  |
| Advise of things prior to the occurrences in a normal progress   - Obtain the permission before conducting any procedure to the woman |  |  |
| Offer womans an opportunity to make suggestions   - Involve the woman by getting her opinion |  |  |
| Extend advices and encouragements   - Involve the woman by getting her opinion |  |  |
| Guide the woman throughout childbirth process and assist with delivery   - Any required assistance |  |  |
| **Emotional Support Activities During Childbirth (midwifery care when woman is responding to pain)** | | |
| Instil confidence to woman by encouraging the presence of companion to help woman through pain,   - Enquire from a woman her preference of a person to be present during labour |  |  |
| Be understanding, friendly and reassuring to the woman   - Be professional and approachable |  |  |
| Encourage free choice on activity, and encouragement of full participation throughout childbirth   - Allow walking, sitting squatting (but take the maternal and fetal condition into consideration) |  |  |
| Encourage the integration of cultural/ personal preferences in the midwifery care   - Consider the preferences of the woman ((but take the maternal and fetal condition into consideration) |  |  |
| Show respect   - Obtain consent - Call by name |  |  |
| **Supportive Care Activities During labour** | | |
| Provision of physical comfort measures, for example, touch and assisting by fulfilling specific request |  |  |
| Attendance of elimination needs |  |  |
| Midwife care for more than one woman at the same time |  |  |

**NB!!!! The informal conversation between the midwife/ woman related to all observations was recorded under the specific theme.**

**VISUAL ANALOG SCALE (VAS) = 100mm**

PARTICIPANT : ________________________

CERVICAL DILATATION : ________________________DATE _______________________

TIME : ________________________

**The Visual Analog Scale (VAS) was given to women and their midwives with the aim of** comparing **the childbirth *pain experienced* among women and the *pain exhibited* by midwives.**

**A woman and her midwife to complete the scale independently during the following phases of cervical dilatation:**

- **0-3 cm**
- **4-7 cm**
- **>8 cm**

**All assessments were completed without reference to the previous rating.**

INSTRUCTION

**Kindly place a mark through the line to indicate the intensity of the painful stimuli.**

**Example of VAS:**

**This information assisted in determining the physical comforting measures rendered by the midwife when the woman was responding to childbirth pain.**
